# Pushing the Limits of MALDI-TOF Mass Spectrometry: Beyond Fungal Species Identification

**DOI:** 10.3390/jof1030367

**Published:** 2015-10-16

**Authors:** Cosmeri Rizzato, Lisa Lombardi, Marina Zoppo, Antonella Lupetti, Arianna Tavanti

**Affiliations:** 1 Department of Biology, University of Pisa, Via San Zeno 37, 56127 Pisa, Italy; E-Mails: cosmeri.rizzato@unipi.it (C.R.); lisa.lombardi@for.unipi.it (L.L.); marina.zoppo@yahoo.it (M.Z.); 2 Department of Translational Research and New Technologies in Medicine and Surgery, University of Pisa, Via San Zeno 37, 56127 Pisa, Italy; E-Mail: antonella.lupetti@med.unipi.it

**Keywords:** MALDI-TOF mass spectrometry, fungal diagnosis, drug resistance, biotyping

## Abstract

Matrix assisted laser desorption ionization time of flight (MALDI-TOF) is a powerful analytical tool that has revolutionized microbial identification. Routinely used for bacterial identification, MALDI-TOF has recently been applied to both yeast and filamentous fungi, confirming its pivotal role in the rapid and reliable diagnosis of infections. Subspecies-level identification holds an important role in epidemiological investigations aimed at tracing virulent or drug resistant clones. This review focuses on present and future applications of this versatile tool in the clinical mycology laboratory.

## 1. Introduction

A rapid and accurate species identification is mandatory for the successful management of fungal infections, the incidence of which has risen over the two past decades. The most common fungal species involved include *Candida* and *Aspergillus* genera [1,2]. However, the scenario of causative agents responsible for both localized and systemic mycoses has changed in the past few years, with emerging species or uncommon fungal species often associated with symptomatic episodes in immunocompromised patients. Among the most significant emerging pathogens are yeast such as *Candida glabrata* [3,4], *Trichosporon* spp. [5] and filamentous fungi including *Scedosporium* spp., *Fusarium* spp., and *Mucorales* [6,7]. In addition, current advances in patient management and the use/abuse of wide spectrum antibiotics has turned invasive fungal infections into a growing concern, especially in the intensive care unit. Although biochemical profile-based and time-consuming microscopy assisted methods have dominated mycology diagnostic laboratories for many years, proper species identification is sometimes hindered by several factors, such as complex taxonomy, genetic relatedness of species, and misleading microscopic evidence. In the past few years matrix-assisted laser desorption ionization time of flight (MALDI-TOF) has revolutionized medical microbiology [8], enabling rapid and accurate bacterial species identification (within a few minutes) with simple and reliable procedures [9]. The technique is based on the identification of characteristic protein patterns derived from microbe composition followed by direct interrogation of updated databases for proper species identification. Since its first application to yeast identification [10], there has been a growing body of evidence indicating that microbial fingerprint by MALDI-TOF can represent a robust and fast tool for routine identification of clinically important fungi [11,12,13,14,15,16,17,18,19]. Subspecies-level identification holds an important role in epidemiological investigations aimed at tracing virulent or drug resistant clones, and it is usually achieved by DNA-based methods such as multi-locus sequence typing (MLST), microsatellite genotyping, amplification fragment length polymorphism (AFLP), DNA microsatellite typing, or pulsed field gel electrophoresis (PFGE) [20,21,22,23,24,25]. These techniques offer very high accuracy and discriminative power; however, they are impractical to perform on a routine basis due to their high costs and time-consuming procedures. The possibility of identifying intra-species clones by MALDI-TOF has been demonstrated for several clinically relevant bacteria, such as methicillin-resistant *Staphylococcus aureus* strains [26], *Haemophilus influenzae* Type b isolates [27] or *Clostridium difficile* [28]. While this seems to be well established for bacteria, the role in sub-species typing of fungi by MALDI-TOF remains to be properly assessed. The purpose of this review is to put together data available for MALDI-TOF in fungal identification and typing, focusing on present and future applications of this versatile tool in the clinical mycology laboratory.

## 2. MALDI-TOF Mass Spectrometry-Based Fungal Identification: The Importance of a Robust Library of Reference Spectra

Routine identification is performed by positioning a small quantity of fungal cells on a target plate, which are overlaid with a matrix containing a solution of α-cyano-4-hydroxycinnamic acid (CHCA) in a mixture of organic solvents. The sample is then air-dried, and during this process all the molecules become embedded in the matrix, and then the target plate is positioned in the mass spectrometer for automated measurement. A defined mass spectrum is obtained, usually encompassing peaks in the 1000 to 30,000 *m/z* range. Raw spectra from the test sample are acquired and compared to a library of reference spectra already listed in the instrument database. Based on this comparison, different algorithms provide identification results, expressed with score values and the interpretative criteria for naming a given species are provided by the different manufacturers. For example, using MALDI Biotyper automation control and the Bruker Biotyper 3.1 software and library (version 3.1.66, encompassing 4613 entries; Bruker Daltonics, Billerica, MA, USA) the identification scores are as follows: a value ≥2.000 indicates species-level identification, scores between 1.700 and 1.999 refer to genus-level identification, and scores below 1.700 are taken as unreliable identification. Instead, Vitek Mass Spectrometry (MS) acquisition using Myla v3.2 middleware provides confidence values based on the similarity between the unknown yeast and every yeast or yeast group in the two database systems (MS-ID version 2.0 IVD and Vitek MS Plus SARAMIS Knowledge Base Version 4.10 RUO, bioMérieux, Marcy l’Etoile, France). Results are displayed in one of three forms: (i) a single identification (confidence value of 60.0% to 99.9%), (ii) a split identification for which a set of possible organisms is displayed, or (iii) no identification when no match is found. The library of spectra is indeed a very critical point for robust species identification of especially for rare or cryptic species [29]. In fact, several reports indicate poor identification of phenotypically indistinguishable species due to the paucity of representative spectra in the database [30,31] or to the need to modify score thresholds for validated identification [32,33]. In our experience, we found that the original Bruker Daltonics BioTyper library database, encompassing quality-controlled entries for more than 3900 microorganisms, was unable to provide acceptable scores when challenged with *Candida metapsilosis* and *Candida orthopsilosis* identification [30]. The development of an additional in-house extended MS library, which included MALDI-TOF MS spectra from additional reference strains, provided correct identification for all *Candida parapsilosis* species complex isolates included in the study [30]. Obtaining high-quality MALDI-TOF MS spectra of fungal pathogens is more challenging than for most bacterial species and requires an optimized standardization of the culture media, extraction, and preparation procedures. It this respect, it has been shown that reliability of yeast identification can be significantly improved by performing pretreatment extraction procedures using formic acid/acetonitrile [34]. Direct spotting sometimes does not provide reproducible results and mass spectra can result differentially homogeneous if generated from material collected from different media [31,34].

## 3. Yeast Identification from Pure Culture

As yeast cells possess a thick cell wall, its disruption requires an additional extraction step compared to bacterial protocols, with some differences related to different MS systems used. In particular, the best preparation method is the same one used for spectra acquisition during the process of database creation, since this will lead to the highest concordance between acquired test spectra and reference spectra in the identification database. In the case of the MALDI Biotyper, the method involves protein extraction, while in the case of the SARAMIS, VITEK-MS, and Andromas systems, on-target-lysis is used [11]. In the “on-target-lysis” technique [35,36], the colony smear is overlaid with 0.5–1 μL 25%–70% formic acid and is allowed to dry before overlaying with the matrix. Ethanol fixation prior to analysis has previously been shown to increase peak numbers [37], probably aiding in cell lysis, and some protocols use absolute ethanol to improve efficiency [38]. In those cases for which on-target-lysis is not sufficient to obtain high quality peaks, cells can also be harvested and washed in 70% ethanol, dried, and then lysed in 70% formic acid, followed by a protein solubilisation step by ACN addition. The debris is removed by centrifugation, and the extract spotted on the target plate. This process generally leads to an increased number of distinct peaks, which give rise to highly discriminating spectra usable for classification in all systems. For cells that still do not lyse efficiently, other extraction methods, such as mechanical disruption in a bead-beater, can be applied [10,18,39].

Other studies focused on the quantity and the age of cultured yeast colonies; Marklein and colleagues reported that it was necessary to have five colonies isolated from CHROMAgar (CHROMagar Microbiology, Paris, France) to provide an accurate identification of yeast isolates [19], while in a successive publication it was determined that an accurate identification can be also achieved by starting from a single colony [35]. Furthermore, different culture time points were also compared in order to determine the influence of cultivation time on accurate identification of fungal pathogens. No significant differences were found when using 24, 48, or 72 h cultures [35].

Many studies have demonstrated that MALDI-TOF MS successfully differentiates yeast isolates [40,41,42] with a similar species identification rate to Bruker and Shimadzu systems (97.6% and 96.1%, respectively [43]) and returns results comparable to those obtained from biochemical tests (96.9%) [43].

MALDI-TOF MS has been proven to efficiently discriminate between closely related species of the *Candida* genus, such as members of the *C. glabrata* clade (*C. glabrata*, *C. nivariensis*, and *C. bracarensis*), members of the “psilosis” complex (*C. parapsilosis*, *C. metapsilosis*, *C. orthopsilosis* and *Lodderomyces elongisporus*), as well as between *C. albicans* and *C. dubliniensis* [44]. Unambiguous identification of most of the above mentioned species groups currently depends on molecular methods, as biochemical ones do not allow for their discrimination [44].

Early reports showed unreliable identification rates for *Cryptococcus neoformans* in comparison with biochemical methods [45], while implementation of database entries assured good identification performance for *C. neoformans* and *C. gattii* species [16]. Additionally, a cost analysis was conducted in which 138 common and 103 archived strains were tested for yeast identification. The analysis determined that MALDI-TOF MS operating costs were lower than those of most conventional and molecular testing systems in terms of reagent costs and hands-on time required for sample processing [46].

Putignani and colleagues analysed spectra obtained by the Bruker BioTyper software from 303 clinical isolates using standard pattern matching. Identifications were compared to identifications by a reference biochemical-based system (Vitek-2 (Biomérieux, Marcy-l’Étoile, France)), and when results were discordant, BioTyper identifications were verified with genotyping identifications obtained by sequencing of the 25S-28S rRNA hypervariable D2 region. Of the 26 discordant results, only five appeared to be real once further determinative testing was performed. The BioTyper showed high analytical performance and was able to discriminate patterns for strain typing of some species [47].

## 4. Mold Identification from Pure Culture

Among fungi, ascomycetous and basidiomycetous yeasts, including *Candida*, *Pichia*, and *Cryptococcus* genera, usually yield highly reproducible spectra. This is mainly due to the fact that these microorganisms uniformly grow on agar plates and are efficiently lysed following the recommended sample preparation protocols [11]. Unfortunately, this does not apply to molds, for several reasons. First, a proper lysis of the cells during the extraction process is partially impaired by a thicker cell wall, together with the articulated morphology that hyphomycetes can acquire. In addition, several factors might have a significant influence on the spectrum profile, including different maturation stages of the selected colonies, the presence/absence of conidia, and agar contamination of the analyte, depending on the extent of agar invasion [48]. An additional issue is the presence of melanin in some molds, which may interfere with ionization [49]. In addition, the choice of the preparation protocol, of the matrix and solvent, and of the pre-analytical steps can introduce a further degree of variation in number and identity of mass peaks obtained [50,51]. Therefore, it is mandatory to take into account all of these sources of variability by testing different preparation techniques in order to construct and then challenge spectrum libraries for diagnostic identification systems. To address this, several efforts have been made in the attempt to standardize growth conditions and sample preparation. At the beginning, conidia forms were selected; however, this led to spectra with low discriminatory power [52]. Moreover, in the case of strongly pigmented isolates, as in the case of *Aspergillus niger* and *Fusarium* spp., the conidial melanin pigment inhibited analyte ionization [50,53]. Suppressing pigment formation by growing the fungus in liquid culture [49] or by performing pre-analytical washing steps [50] can help overcome this problem. Generally, better spectra can be obtained from liquid culture or by directly scraping micelial cells from agar plates [18,54]. Although the on-target-lysis performed with acid-containing matrix solution might be sufficient for many molds, mechanical disruption through bead-beating of cells and conidia prior to analysis is advisable for *Penicillium* and *Aspergillus*, leading to highly discriminating spectra [18,39,54]. Since a unique standardised protocol is not yet defined, a major role in addressing the heterogeneity in the clinical diagnosis of molds is played by the database used for identification, which is based on the peaks observed. Indeed, an additional way to address this challenge is to set up the database so that it contains multiple entries for each species [55].

In this respect, Norman and colleagues assessed the influence of several parameters on identification effectiveness. The parameters included: (i) raw spectra used to build a reference mass spectrum database (RMS); (ii) reference mass spectra included per strain; and (iii) strains per species included in the library on identification effectiveness.

The main results obtained indicated that incorporating an increased number of subcultures from each strain and increasing the number of strains representing each species are key to improving the architecture of RMS libraries. These findings should be taken into account for the construction of a more effective library in clinical laboratories [56].

This is the case of VITEK-MS and Andromas databases [57,58], which contain multiple spectra for each species to encompass differences in the observed peaks due to variable growth conditions. In the SARAMIS system, for each species, a “super-spectrum” is available that contains peaks that can be consistently observed irrespective of the growing condition [57]. An opposite strategy has been pursued by the MALDI Biotyper system, which provides the user with an additional library of spectra acquired after filamentous fungi are grown in Sabouraud liquid broth, thus standardising this crucial step (“Fungi library”, Bruker Daltonics). Despite the additional complexity linked to the previously mentioned factors, once a species is included in the database with multiple entries, clinical isolates can be successfully identified. An example is represented by *Fusarium* spp., which has emerged as a significant human pathogen causing both superficial and life-threatening invasive infections, mainly in immunocompromised patients [59]. Unfortunately, this genus is one of the most difficult to identify by conventional macroscopic and microscopic examination, due to the duality between sexual and asexual stage taxonomy and its high phenotypic variability [60]. MALDI-TOF MS has been proven as a reproducible and robust method for the identification of clinical isolates belonging to different *Fusarium* species, including *Fusarium solani*, *F. oxysporum*, *F. verticilloides*, *F. proliferatum*, and *F. dimerum*, only failing in the case of species not represented in the reference database [32]. A rapid identification is important not only for epidemiological purposes, but also for therapeutic treatment, since isolates belonging to the *F. solani* complex are significantly less susceptible to voriconazole and posaconazole compared to the other species [61,62]. Identification by MALDI-TOF MS can also be carried out for dermatophytes, filamentous fungi classified in *Microsporum*, *Tricophyton*, and *Epidermophyton* genera. Once again, a key factor is the media used, which may or may not delay sporulation. In most cases, heterogeneous spectral profiles obtained from culture at different growth stages also allow the separation between different species, although not the identification of cryptic ones [31]. MALDI-TOF MS results showed that this method is a reliable tool for dermatophyte diagnostics, with a percentage of correct identification at the species level of up to 95.8% [63,64].

## 5. MALDI-TOF MS Identification from Clinical Samples

Fungal systemic infections are on the rise, in part because of advanced treatment strategies for chronic degenerative pathologies, which have led to an increase in the number of immunocompromised patients [65]. Systemic candidiasis has a high mortality rate especially if associated with septic shock, rising to up to 97.6% if these patients are not treated with tailored antifungal therapy within 24 h [66]. The possibility of a rapid identification of fungal species directly from clinical samples and a contextual evaluation of drug susceptibility without the need of sub-cultured isolates for testing would significantly speed up the classical workflow and provide a reduced time frame for results. In this respect, MALDI-TOF MS has been demonstrated to be able to provide an early diagnosis of yeast species from positive blood cultures, bypassing the subculturing step and leading to reductions in turnaround time. This is potentially beneficial for the patient, even though outcomes may vary depending upon the different protocols adopted [67,68,69,70,71,72]. A recent paper by Idelevich and coworkers investigated the usefulness of a combined approach in which very short-term blood cultures are used for MALDI-TOF MS identification and are coupled with an early inoculation of an automated susceptibility testing device from the same biomass [73]. The authors were able to confirm the ability of mass spectrometry to identify different yeast species when directly applied to early positive blood cultures, even though the success rate was strongly dependent upon fungal concentration in the sample, as also reported by other investigators [72]. However, when yeast cell pellets prepared with the same standard Sepsityper kit protocol were used for antifungal susceptibility testing in a combined effort to provide an early response for drug susceptibility, performance was sub optimal. In fact, while amphotericin B, flucytosine, and caspofungin gave 100% consensus with standard susceptibility testing methods, azole failed to yield a positive outcome [73]. In addition, for some of the isolates the test was aborted, most likely due to the paucity of fungal cells in the direct inoculum, clearly indicating that testing from subcultured isolates is still required and should flank direct testing at least for validation of the results [73].

It has been shown that MALDI-TOF MS can be applied to the direct and accurate identification of bacteria from the majority of urine samples with a colony number higher than 10^5^ colony forming units (CFU)/mL, coupled with flow cytometry as a screening test [74]. Urinary infections are very common in the nosocomial environment, with one of the major risk factors being the use of catheterization procedures [75]. The most frequent causative agent is *Escherichia coli* and Enterobacteriaceae in general, followed by other Gram-negative bacteria, such as *Pseudomonas* spp. and *Acinetobacter* spp., Gram-positive cocci, and, among fungi, *Candida* species [76,77]. Kim and colleagues recently compared MALDI-TOF MS and conventional biochemical testing in the identification of the most relevant uropathogens directly from urine specimens, including specimens with yeast [78]. While they obtained high accuracy for Gram-negative bacteria, their findings were less encouraging for Gram-positive and yeast containing samples. In fact, a low identification rate (20%) was observed for yeast, with the Vitek MS showing superior ability of identification of yeasts grown on agar [78]. This highlights the need for an optimized pre-treatment step, which could eventually improve the method outcome and lead to a potential direct identification of fungal urinary infections.

A recent report by Sendid and colleagues describes an innovative MALDI-TOF MS-based approach to identify a specific signature of bloodstream infections caused by *C.* *albicans* [79]. This would be particularly important, as one of the major issues in the management of these infections is related to the difficulty of obtaining an early diagnosis, which is mandatory for a favourable outcome. The approach described by Sendid *et al.* takes inspiration from previously available tests aimed at detecting *Candida* cell wall moieties (e.g., mannans, glucans) by immunocapture or biochemical techniques in sera of patients with candidemia [80]. Following optimization of pre-analytical procedures aimed at extracting oligosaccharides from serum, mass spectrum analysis revealed a peak of *m/z* 365, which was identified as a disaccharide. Interestingly, this disaccharide was specifically associated to sera obtained from patients with candidaemia and was absent in control sera collected from healthy individuals. This finding was elegantly confirmed by using murine models of *C. albicans* colonization and infection, indicating that this biomarker was specifically associated with systemic infection, and highlighting the potentiality, if confirmed, of this mass spectrometry-based method for rapid discrimination between colonized individuals and patients with disseminated candidiasis [79].

## 6. Intraspecific Subtyping

One of the main areas of current research in the management of mycoses is intraspecific typing, which plays an important role in epidemiological investigations aimed at establishing relatedness among strains and at tracing virulent or drug resistant clones. Several authors have investigated the potentiality of MALDI-TOF MS in subspecies-level identification. Attempts were first made with bacteria, and successful MALDI-TOF MS typing examples have been reported for methicillin-resistant *Staphylococcus aureus* strains [26], *Arthrobacter* [81], *Salmonella* [82], and *Legionella* strains [83].

### 6.1. Detection of Antifungal Resistance

The potential application of MALDI-TOF MS for the detection of fungal antimicrobial resistance is a field that is of paramount epidemiological importance. In 2009, Marinach and colleagues monitored the proteome of *Candida albicans* grown in the presence of different concentrations of fluconazole by MALDI-TOF MS [84]. A careful standardization of the protocol used to perform the assay led to the identification of a “minimal profile change concentration”, which defined the lowest fluconazole concentration inducing a mass spectrum profile detectable by the instrument. The panel of fluconazole-resistant strains of *C. albicans* was allocated to the correct category as defined by the Clinical and Laboratory Standards Institute (CLSI), regardless of the molecular mechanism/s of fluconazole resistance or genetic background [84]. Saracli and co-workers, who recently described the ability to detect triazole resistance by MALDI-TOF MS in *C. albicans*, *C. tropicalis*, and *C. glabrata* isolates, used a similar approach. This study included a panel of resistant, susceptible dose-dependent, and susceptible strains for each of the species included in the study as established by the reference CLSI method. Reproducible positive results ranged between 54.3% and 82.9%, with best performance obtained for *C. glabrata*, followed by *C. albicans*. Scarce reproducibility was obtained for *C. tropicalis* strains, thus indicating that further analysis and protocol optimization are still required prior to applying this technique as an alternative to conventional antifungal susceptibility testing [85]. MALDI-TOF MS methodology was also applied to test caspofungin susceptibility of wild-type and FKS mutant isolates of *Candida* and *Aspergillus* species, with the aid of a composite correlation index approach, based on the same concept reported above (minimal profile change in concentration induced by caspofungin at different concentrations) [86]. The results obtained indicate that this technique correctly detects *Candida* and *Aspergillus* isolates with a glucan synthase mutation, suggesting that a significant variation of proteome induced by caspofungin can be taken as a predictive value for echinocandin resistance [86,87,88]. Therefore, even if further studies are still needed to ascertain the real potential applicability in the evaluation of antifungal susceptibility, this technique holds the promise of becoming a versatile and widely exportable approach and today stands as a valid aid to the effective detection of clinical isolates with a reduced antifungal susceptibility profile.

### 6.2. Genotyping vs. MALDI-TOF MS Clustering

Over the past two decades, DNA-based molecular typing of fungal isolates responsible for nosocomial infections has significantly contributed to an improved understanding of the epidemiology of mycoses. However, most DNA techniques are costly, time consuming, and require trained personnel. The ability of MALDI-TOF MS to discriminate *C. albicans* clinical isolates has been demonstrated in 2008 [37]. In a recent paper, Dhieb and colleagues [89] compared Microsatellite Length Polymorphism (MLP) typing with MALDI-TOF clustering analysis of 102 *C. albicans* clinical isolates. Their findings indicate that MALDI-TOF MS-generated clusters failed to reliably recognize genetically related *C. albicans* isolates. Conversely, when MLP analysis was compared to mass spectrum phenotyping in 58 *C. glabrata* clinical isolates collected from different geographical areas, most of the isolates were clustered by MALDI-TOF according to their MLP genotype, with a few exceptions. This indicates that two different clustering approaches, one based on DNA differences and the other on reflection changes in the protein spectrum, were successful in determining the population structure of *C. glabrata*, which was related to geographic origin or other phenotypic properties [90]. Dendrograms generated by microsatellite analysis and MALDI-TOF were also congruent in the case of a *C. parapsilosis* outbreak in a neonatal intensive care unit [91]. In our laboratory, we compared the discriminatory power of MALDI-TOF MS with the DNA based Amplification Fragment Length Polymorphism (AFLP) for intra-specific typing of clinical isolates belonging to the *C. parapsilosis* species complex [30]. In particular, mass spectra obtained from a panel of independent isolates as well as from a set of sequential isolates from a single patient were clustered by the BioTyper 3.0 software (Bruker Daltonics), and MALDI-TOF MS-generated dendrograms were compared to those obtained by AFLP. Hierarchical cluster analysis of main spectra profiles of *C. parapsilosis*, *C. orthopsilosis*, *C. metapsilosis*, and *L. elongisporus* correctly allocated isolates into separate groups, according to their species designation, which was in agreement with the UPGMA dendrogram generated from AFLP profiles. However, the dendrogram derived from protein profiling showed intra-species distances between isolates that differed from those obtained in AFLP-based dendrogram, particularly with regard to *C. metapsilosis* isolates. Isolates belonging to the latter species were in fact characterised by very little mass spectrum diversity. Our findings indicate that AFLP remains the preferred typing method for intra-species correlation, due to its higher discriminative power. It is plausible that the discrepancies observed in strain relatedness obtained with the two techniques reflect the different nature of the approaches used [30].

### 6.3. Proteomic Fingerprinting in Association with Other Clinically Relevant Features

The potential association of MALDI-TOF spectra with clinically relevant phenotypic traits other than drug resistances, such as virulence or biofilm production, may also contribute to the improvement of the therapeutic management of fungal infections. Biofilm formation is considered an important feature in clinical settings, since it is associated with the colonization of prosthetic material and it is known to act as an efficient barrier against antifungal drugs and/or the immune system [92]. The possibility of knowing if the infecting strain is a biofilm producer could provide important information on the infection prognosis and could also influence the choice of antifungal regimen. In this regard, Kubesova and co-workers described the use of MALDI-TOF MS to discriminate *C. parapsilosis*, *C. metapsilosis*, and *C. orthopsilosis* biofilm producer isolates from non-producers. MALDI fingerprints of biofilm-positive isolates were characterized by specific peaks that could not be detected in biofilm-negative isolates [93]. Although these data were obtained using a limited number of isolates, their findings were confirmed by two-dimensional gel electrophoresis, pointing to a potential application of MALDI-TOF phenotyping in clinical settings.

## 7. Conclusions

In a scenario where disseminated fungal infections by emerging pathogens are on the rise, the need for a rapid and accurate diagnosis is mandatory in order to achieve positive treatment outcomes. Species identification by Matrix-Assisted Laser Desorption Ionization-Time of Flight Mass Spectrometry already represents a reality in the routine clinical laboratory, even though databases need to be continuously updated to include reference spectra of unusual pathogens and protocols for a direct application from clinical samples standardized. Implementation of MALDI-TOF MS for intra-specific typing holds great promise, as it has demonstrated the ability to detect drug resistant phenotypes, biofilm-producing strains, and potentially cluster isolates for epidemiological purposes. Though some aspects of biotyping protocols based on MS still require standardization/validation, today these applications seem more feasible and in most cases one step away from a direct role in the fight against fungal infections.
